# Biomarkers in axial spondyloarthritis diagnosis: from clinical signs to multi-omics integration

**DOI:** 10.3389/fimmu.2026.1929862

**Published:** 2026-09-07

**Authors:** Peijin Xin, Zi Xu, Yuanyi Pan, Mudan Zhang, Rongpin Wang

**Affiliations:** Guizhou Province International Science and Technology Cooperation Base for Precision Imaging Diagnosis and Treatment, Key Laboratory of Advanced Medical Imaging and Intelligent Computing of Guizhou Province, Department of Radiology, Guizhou Provincial People’s Hospital, Guiyang, China

**Keywords:** axial spondyloarthritis, biomarkers, diagnosis, multi-omics, radiomics

## Abstract

Axial spondyloarthritis (axSpA) is a chronic inflammatory rheumatic disease primarily affecting the sacroiliac joints and spine, with an estimated global prevalence of 0.3–1.5%. Despite advances in classification criteria and imaging techniques, diagnostic delay remains a persistent clinical challenge, with patients waiting a median of 2–6 years from symptom onset to confirmed diagnosis. Biomarkers—broadly categorized into clinical, molecular, and imaging modalities—offer the potential to shorten this diagnostic gap by providing objective, quantifiable, and earlier indicators of disease. This narrative review maps the current landscape of biomarkers for the diagnosis of axSpA across all three modalities, with particular attention to how machine learning and multi-omics integration are reshaping the diagnostic paradigm. We examine established clinical markers (HLA-B27, inflammatory back pain criteria, C-reactive protein), survey the molecular biomarker literature spanning genomics, proteomics, metabolomics, and liquid biopsy, and trace the imaging trajectory from conventional radiography and MRI through to radiomics and deep learning models that now approach the performance of expert radiologists. We further explore multimodal fusion—the integration of clinical, molecular, and imaging biomarkers into unified diagnostic models—and identify barriers to clinical translation: the lack of prospective external validation, inconsistent standardization, and unresolved questions about algorithmic fairness across diverse populations. We conclude that biomarker-driven precision diagnosis in axSpA requires continued biomarker discovery alongside the construction of shared infrastructure—multi-modal datasets, standardized pipelines, and prospective validation cohorts—to integrate existing and emerging biomarkers into clinically deployable tools.

## 1 Introduction

Axial spondyloarthritis (axSpA) is a chronic inflammatory disease predominantly affecting the axial skeleton, with sacroiliac joint involvement as its hallmark feature. Global prevalence estimates range from 0.3% to 1.5%, with substantial geographic and ethnic variation that mirrors the population distribution of the human leukocyte antigen B27 (HLA-B27) allele (1, 2). The disease encompasses a spectrum from non-radiographic axSpA (nr-axSpA)—where structural damage is not yet visible on conventional radiography—to radiographic axSpA, also termed ankylosing spondylitis (AS), characterized by definitive sacroiliitis on X-ray.

The diagnostic journey for patients with axSpA remains protracted. A 2022 systematic review of 69 studies reported median diagnostic delays ranging from 0.67 to 8 years globally, with most studies reporting delays between 2 and 6 years (1). These median figures, though sobering, understate the true burden: mean delays, which are inflated by long-tail outliers, have been reported as high as 6.7 years in some meta-analyses (3). The diagnostic delay in axSpA is multifactorial: early back pain is often nonspecific. It lacks simple disease-specific biomarkers, awareness among patients and physicians remains low, and misdiagnosis as mechanical disorders is common. Diagnostic delay may be particularly pronounced in women and in patients facing socioeconomic barriers (1, 2, 4, 5). Encouragingly, temporal trends suggest improvement—a 2024 analysis of the Brazilian Spondyloarthritis Registry documented a decline in mean diagnostic delay from 41.7 years in the 1960s to 1.9 years during 2020–2024 (6). Yet even at the lower bound of current estimates, a delay of approximately two years between symptom onset and diagnosis means that many patients have already accrued irreversible structural damage at the point of treatment initiation.

Central to the diagnostic challenge is the nature of the tools at the clinician’s disposal. The Assessment of SpondyloArthritis International Society (ASAS) classification criteria, published in 2009, were a landmark advance in recognizing early axSpA before radiographic damage becomes apparent (7). The criteria employ a two-arm design: an imaging arm (sacroiliitis on MRI or definite radiographic sacroiliitis plus at least one SpA feature) and a clinical arm (HLA-B27 positivity plus at least two other SpA features). Yet significant limitations persist. The sensitivity of the imaging arm depends critically on MRI-detected bone marrow edema (BME), which is absent in up to 30% of HLA-B27-positive patients with convincing clinical axSpA (8). The clinical arm, in turn, is heavily dependent on HLA-B27 status—a marker whose prevalence varies more than tenfold across ethnic groups, from approximately 8% in Caucasian populations to approximately 1% in Japanese populations (9). Furthermore, the ASAS criteria were designed for research classification, not individual clinical diagnosis, and their application in low-prevalence primary care settings can produce false positives that erode diagnostic confidence (8).

The biomarker landscape across axSpA diagnosis has expanded substantially over the past decade. Existing reviews, however, have largely examined individual biomarker classes in isolation—imaging biomarkers (10), genetic markers (11), or proteomic signatures (12)—without placing them within a unified framework. This fragmentation obscures a critical insight: the diagnostic limitations of any single biomarker modality are, in principle, addressable through integration. A patient with an equivocal MRI may harbor a revealing molecular signature; a patient who is HLA-B27-negative may have imaging features that resolve the diagnostic ambiguity. The convergence of radiomics, ML, and multi-omics technologies creates, for the first time, a realistic pathway toward such integration.

**Figure 1:**
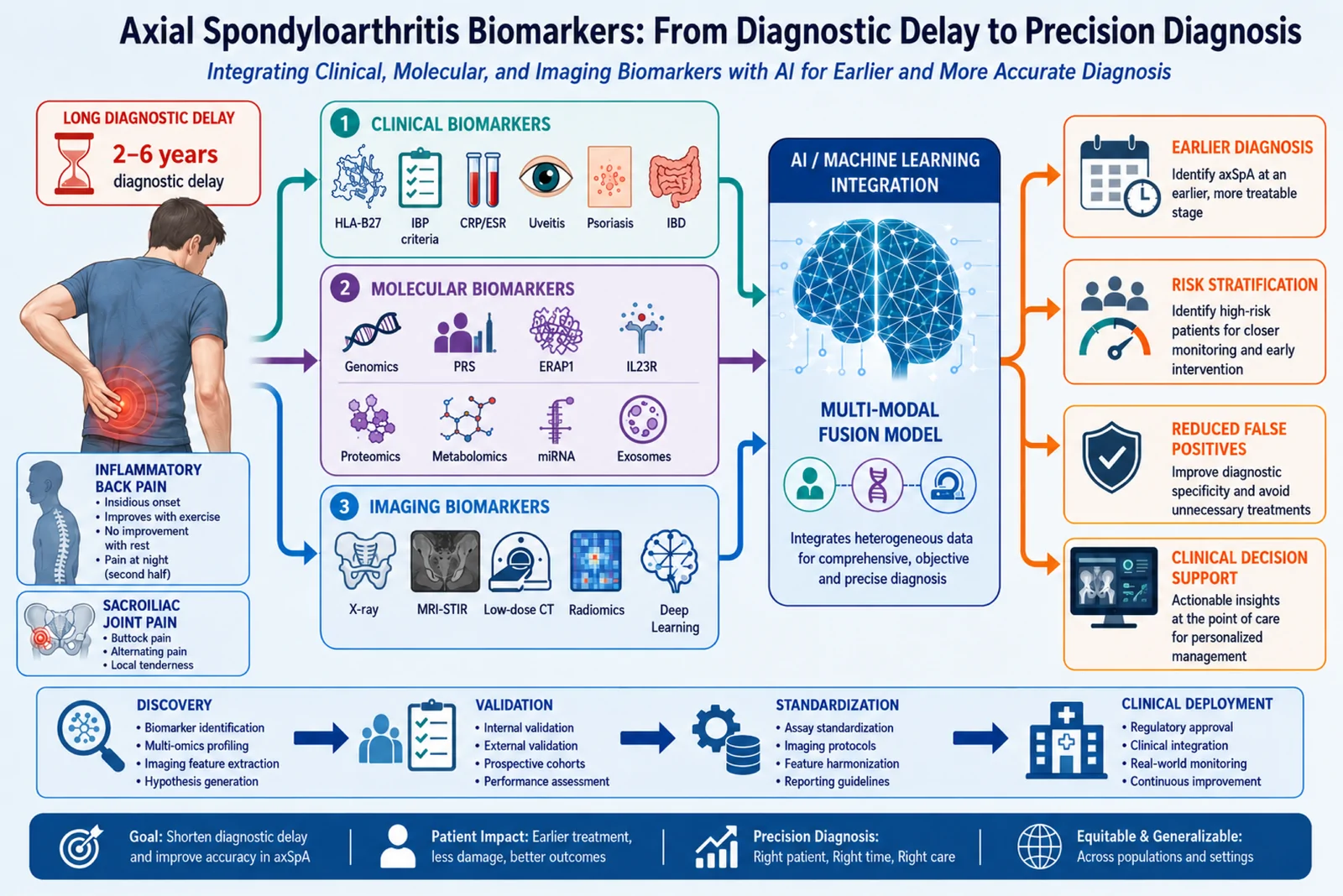
Integrating clinical, molecular, and imaging biomarkers with AI for earlier and more accurate diagnosis.

This review surveys the current state of biomarker research for axSpA diagnosis across three interconnected domains: clinical biomarkers (Section 2), molecular biomarkers (Section 3), and imaging biomarkers (Section 4), and then moves to AI-driven radiomics and deep learning (Section 5). Section 6 examines the nascent but rapidly evolving field of multi-modal integration, and Section 7 identifies the critical challenges of validation, standardization, clinical deployment, and fairness that must be addressed before biomarker-driven precision diagnosis can enter routine clinical practice. **Figure 1** provides an graphic overview of this review.

### 1.1 Review methodology

A structured literature search was conducted in PubMed, Embase, and Web of Science for English-language publications between January 2015 and December 2025, using combinations of the terms “axial spondyloarthritis,” “ankylosing spondylitis,” “biomarker,” “diagnosis,” “HLA-B27,” “genomic,” “proteomic,” “metabolomic,” “imaging,” “MRI,” “radiomics,” “deep learning,” “machine learning,” “artificial intelligence,” “multi-omics,” and “multi-modal.” Additional sources were identified through citation tracking of seminal papers and targeted searches of arXiv, bioRxiv, and medRxiv for recent AI/radiomics preprints. Inclusion criteria comprised original research, systematic reviews, and meta-analyses focusing on diagnostic biomarkers in adult (≥18 years) populations with axSpA. Studies limited to juvenile/enthesitis-related arthritis, purely prognostic or treatment-response biomarkers without diagnostic relevance, case reports, and editorials were excluded. Reference screening was performed by a single reviewer using a narrative synthesis approach. This methodology follows the Scale for the Assessment of Narrative Review Articles (SANRA) framework, while acknowledging that a narrative, rather than a systematic, review design was adopted given the breadth and heterogeneity of the biomarker modalities surveyed.

## 2 Clinical biomarkers: the diagnostic foundation

### 2.1 HLA-B27 — the cornerstone with cracks

HLA-B27 remains the single most informative clinical biomarker for axSpA diagnosis, present in 80–95% of AS patients of European ancestry and 70-80% of the Chinese population (9, 13). Its clinical utility, however, is bounded by three constraining facts. First, the prevalence of HLA-B27 in the general population varies dramatically by ethnicity: approximately 8% in Caucasians, 4–8% in Han Chinese, 2% in African Americans, and approximately 1% in Japanese populations, following a north-to-south gradient that mirrors AS prevalence (9). Second, only 5–8% of HLA-B27-positive individuals in the general population ever develop AS, thereby limiting the marker’s positive predictive value for population screening. Third, not all HLA-B27 subtypes confer risk: B27:05 and B27:04 are strongly associated with AS, whereas B27:06 and B27:09 appear to confer little or substantially lower disease risk in the populations in which they are most observed (11). In African American AS patients, only approximately 48% test positive for HLA-B27, substantially limiting its utility as a rule-out test in this population (9). These ethnic and subtype-level variations underscore a core principle: HLA-B27 cannot serve as a universal, standalone diagnostic tool and must be interpreted within the context of pre-test probability, clinical presentation, and population-specific prevalence.

### 2.2 Clinical phenotypes as diagnostic signals

Inflammatory back pain (IBP) is the sentinel clinical feature of axSpA, and its characterization has been formalized through successive criteria sets. The Calin criteria of 1977 offer high sensitivity (76–91%) but limited specificity (60–75%), making them suitable for screening (14). The Berlin criteria of 2006, particularly the 8a set, achieve the highest specificity (82–94%) and positive likelihood ratio (12.0) (15). The ASAS expert criteria of 2009 provide a balanced approach but do not clearly outperform their predecessors in head-to-head comparisons (16). The practical implication is that IBP criteria are most useful when applied sequentially—using Calin for screening and Berlin for confirmation—rather than as isolated diagnostic tests.

Peripheral and extra-articular manifestations provide additional diagnostic clues. Enthesitis affects 30–50% of axSpA patients, peripheral arthritis approximately 30%, and dactylitis approximately 6% (17). Anterior uveitis, occurring in 25–30% of AS patients, is a particularly informative extra-articular feature, and its presence in a patient with chronic back pain should prompt a high index of suspicion for axSpA. Psoriasis and inflammatory bowel disease are present in approximately 10% and 5–10% of axSpA patients, respectively, and their recognition can anchor the diagnosis within the broader spondyloarthritis spectrum (17).

### 2.3 Laboratory inflammatory markers and composite indices

Conventional inflammatory markers—C-reactive protein (CRP) and erythrocyte sedimentation rate (ESR)—exhibit a well-documented ceiling in axSpA diagnosis. CRP is elevated in only 40–50% of axSpA patients overall, and the proportion drops to 20–30% in nr-axSpA, where the absence of radiographic damage already makes diagnosis challenging (13). The Bath Ankylosing Spondylitis Disease Activity Index (BASDAI) and the Ankylosing Spondylitis Disease Activity Score (ASDAS-CRP) are primarily disease activity monitoring tools rather than diagnostic instruments, though they can contribute to the overall clinical picture when interpreted alongside imaging and genetic data (18).

### 2.4 Sex differences

Sex constitutes an important but underappreciated modifier of the axSpA diagnostic pathway. A 2024 meta-analysis of 26 studies (21,704 patients) found that women experienced a mean diagnostic delay 1.48 years longer than men (95% CI 0.83–2.14; p < 0.0001), widening to 3.16 years in extra-European countries. Women more often present with nr-axSpA, distinct pain patterns, and greater enthesitis and fibromyalgia comorbidity (13.1–50% vs. 0–13.1% in men)—all of which confound the clinical picture (2). Encouragingly, the sex gap in diagnostic delay has narrowed since the introduction of the ASAS criteria in 2009, suggesting that MRI-based detection of early disease has partially mitigated sex-related diagnostic disparities (19).

## 3 Molecular biomarkers: beyond HLA-B27

### 3.1 Genetic susceptibility beyond the MHC

Genome-wide association studies have expanded the genetic architecture of AS/axSpA beyond the MHC region, identifying over 100 susceptibility loci involved in antigen processing, immune activation, cytokine signaling, and bone-related pathways (11). Among non-MHC genes, ERAP1 shows the strongest association, with risk variants conferring susceptibility specifically in HLA-B27-positive individuals, supporting a role for altered antigen processing in immune dysregulation (20–23).

Other replicated loci, including IL23R, IL12B, TYK2, STAT3, RUNX3, TBX21, TNFRSF1A, and PTGER4, converge on the IL-23/IL-17 axis, T-cell differentiation, TNF/NF-κB signaling, and prostaglandin-mediated inflammation (23–25). These findings align with therapeutic evidence: TNF and IL-17A inhibitors are effective in axSpA, whereas IL-23 blockade has shown limited benefit in established disease, suggesting stage- or tissue-dependent roles (26–28). Genetic loci such as PTGER4 and RUNX3, along with Wnt/BMP pathway genes, further link inflammation to pathological bone remodeling (29, 30).

Most non-MHC variants confer modest individual effects and are not sufficient as standalone diagnostic biomarkers. Polygenic risk scores aggregating multiple loci have demonstrated improved discrimination over HLA-B27 alone in some populations (AUC 0.92–0.95) (31). However, clinical translation is limited by poor cross-ethnic portability—PRS trained on European cohorts perform less well in East Asian, African, or admixed populations. Thus, cross-ethnic validation and integration with clinical and imaging features are essential before routine use.

### 3.2 Gene expression and cellular omics

Bulk transcriptomic studies of peripheral blood and immune-cell subsets have identified inflammatory programs involving TNF, IL-17-related pathways, innate immune activation, and antigen presentation in axSpA (32, 33). Cellular profiling indicates that axSpA is driven by a multicellular network rather than a single lineage, involving Th17-like cells, MAIT cells, γδ T cells, cytotoxic CD8^+^ T cells, and activated myeloid populations (25, 33–35). MAIT and γδ T cells contribute to IL-17 production, while entheseal stromal cells and mesenchymal progenitors may amplify inflammation and couple it to pathological bone remodeling (29, 30). Single-cell RNA sequencing in axSpA is still emerging. Available tissue-based studies nevertheless suggest a multicellular inflammatory network involving T cells and tissue-resident immune/stromal compartments that may sustain IL-17-dominant inflammation (36–38). However, direct single-cell profiling of sacroiliac joint and spinal entheseal lesions remains limited.

Although scRNA-seq studies in axSpA remain limited, transcriptomic signatures have shown some translational potential. For example, a two-gene signature (KAT2B+EMP3) has been reported to predict rapid radiographic progression, whereas other analyses have proposed immune-related candidates, such as CX3CR1 and PANoptosis-associated genes (36, 39). However, these findings require further validation.

### 3.3 Cytokines, acute-phase proteins, proteomics, and bone remodeling markers

Cytokines and inflammatory proteins remain the most studied molecular biomarkers in axSpA. TNF-α, IL-17A, IL-6, IL-23, and GM-CSF are all linked to inflammatory activity and reflect the central role of the IL-23/IL-17 axis (25–28). IL-17A promotes the production of IL-6, CXCL8, G-CSF, and MMPs, bridging inflammation and tissue remodeling (25, 27, 30).

Conventional acute-phase markers such as CRP and ESR are commonly used but have limited sensitivity, with some patients showing normal CRP despite evident MRI-confirmed inflammatory activity (40). S100A8/A9 correlates significantly with disease activity, MRI inflammation, and radiographic progression (41–43). Soluble CD14 (sCD14) is elevated and correlates positively with ASDAS and CRP (41). MMP-3 is associated with disease activity and structural progression (44). Comparative studies indicate that IL-17/IL-23 cytokines are not the strongest individual discriminators—MIF, MCP-1, and IgA anti-CD74 antibodies showed superior standalone performance (45), and IL-17A correlates only weakly with disease activity and varies by clinical phenotype (46). High-throughput platforms (Olink and SomaScan) have identified TNF, FKBPL, MAPK14, IL-7R, and IL-23R as leading proteomic candidates for axSpA (47). TNF is a circulating mediator, FKBPL and MAPK14 are mainly intracellular pathway-related proteins, whereas receptor-related signals such as IL-7R and IL-23R may reflect soluble receptor variants or pathway activation. Their biomarker utility thus varies; some reflect systemic inflammation, others cellular activation rather than stable circulating markers (48, 49).

Bone remodeling markers—including cartilage degradation markers (COMP, CTX-II), collagen turnover markers (C1M, C2M, C3M), bone formation markers (BALP, osteocalcin, PINP), bone resorption markers (CTX-I, TRACP-5b), and Wnt pathway regulators (DKK1, sclerostin)—indicate the dynamic imbalance among matrix degradation, reparative responses, and aberrant osteogenesis (29, 50). These markers are more strongly linked to structural progression than to early diagnosis, and their most plausible role lies within integrated models that combine inflammatory, genetic, clinical, and imaging data.

### 3.4 Metabolomics and the gut-joint axis

Metabolomic profiling in axSpA has revealed alterations in serum/plasma; tryptophan metabolites via the kynurenine pathway are generally elevated, while levels of short-chain fatty acids such as butyrate and propionate—primarily measured in stool—are reduced in axSpA patients compared with healthy controls. Phospholipid and sphingolipid species are also altered in serum, with some species elevated in association with NF-κB activation (51–54). Tryptophan metabolism may link the gut microbiota and mucosal immunity to the IL-23/IL-17 axis, while SCFAs (e.g., butyrate, propionate) modulate epithelial barrier function and Treg responses. Lipid metabolism is also relevant: PTGER4, a prostaglandin E2 receptor, is an established axSpA susceptibility locus (23).

Metabolic reprogramming may sustain inflammation: Th17 and myeloid cells rely on glycolysis, whereas Tregs depend on oxidative phosphorylation; changes in lactate and TCA intermediates may reflect both immune activation and alterations in the bone microenvironment (25, 30, 54).

Despite its biological plausibility, axSpA metabolomics remains in the discovery phase. Findings vary by biospecimen, platform, treatment, and diet, and no metabolomic marker has been incorporated into clinical guidelines due to cost, accessibility, standardization issues, and insufficient evidence of added value over HLA-B27, CRP, and MRI (55). Currently, metabolomics is best viewed as a tool for exploring the gut-joint axis and generating candidates for future multi-omic panels.

### 3.5 Liquid biopsy: circulating nucleic acids, extracellular vesicles, and epigenetic markers

Liquid biopsy—encompassing circulating miRNAs, lncRNAs, circRNAs, cfDNA, DNA methylation signatures, and extracellular vesicle cargo—offers a noninvasive approach to capture immune activation, epigenetic regulation, and intercellular communication in axSpA.

Circulating miRNAs are the most studied candidates. A recent study reported reduced levels of miR-146a, miR-155, and miR-29a in axSpA compared with controls, with individual AUCs of 0.78–0.85 and a three-miRNA panel AUC of 0.91; miR-155 has also been linked to SOCS3 modulation (56). However, findings vary by sample type, normalization, and disease stage. LncRNAs/circRNAs (H19, MEG3, MALAT1, TUG1, hsa_circRNA_001544, hsa_circRNA_102532) have been implicated in inflammatory/osteogenic pathways but remain preliminary (49, 57, 58). Extracellular vesicles carry distinct miRNA and surface marker profiles in axSpA, though standardization remains challenging (59).

Epigenetic markers may bridge genetics and environment: risk SNPs in axSpA are enriched in enhancer regions near IL23R, RUNX3, and TYK2, suggesting cell-type-specific regulatory effects (60). cfDNA and DNA methylation have been proposed as monitoring tools, but evidence in axSpA is limited (61, 62).

Overall, liquid biopsy biomarkers are promising but not clinically mature. Most studies are discovery-phase, with small sample sizes and limited validation. Standardization and independent cohort validation are needed before clinical use.

### 3.6 From single molecules to multi-analyte panels

**Table 1** compares the aforementioned molecular biomarkers. A major conclusion across molecular biomarker studies in axSpA is that single markers are unlikely to be sufficient for routine clinical use. HLA-B27, CRP, cytokines, proteomic markers, metabolomic signals, and liquid biopsy candidates each capture only part of a complex disease process. For this reason, the field is moving toward multi-analyte panels that combine genetic, transcriptomic, proteomic, metabolic, and clinical information. Such models may provide better diagnostic or prognostic performance than individual markers, especially when supported by machine learning methods (12, 47). The main challenge is now validation rather than discovery. Future studies should use prospectively collected, multicenter cohorts and demonstrate added value beyond existing tools such as HLA-B27 and CRP.

**Table 1:** Comparison of different molecular biomarkers.

Biomarker type	Main examples	Main value	Key limitation
Genetics	HLA-B27, ERAP1, IL23R, IL12B, TYK2, STAT3, RUNX3, PTGER4, Wnt/BMP genes	HLA-B27 supports diagnosis; other genes reflect immune regulation and bone remodeling.	HLA-B27 is not sufficient for diagnosis; genetic effects vary among ethnic groups.
Transcriptomics	TNF/IL-17 signatures; Th17, MAIT, γδ T, CD8^+^ T, and myeloid cells, KAT2B+EMP3	Reflect the multicellular inflammatory network and may predict progression.	Limited tissue studies and validation.
Proteomics	TNF-α, IL-17A, S100A8/A9, sCD14, MMP-3, MIF, MCP-1; COMP, CTX-II, PINP, DKK1	Assess inflammation, disease activity, and structural progression.	No single marker is sufficiently sensitive or specific.
Metabolites	Kynurenine metabolites, butyrate, propionate, phospholipids, sphingolipids, lactate	Reflect the gut-joint axis and immune metabolic changes.	Influenced by diet, treatment, and detection methods.
Liquid biopsy	miRNAs, lncRNAs, circRNAs, extracellular vesicles, cfDNA, DNA methylation	Offer noninvasive options for diagnosis and monitoring.	Mostly exploratory and not yet clinically validated.

## 4 Conventional imaging biomarkers

### 4.1 Conventional radiography: the historical standard and its limits

Pelvic radiography has been the first-line imaging modality for suspected sacroiliitis for decades, codified in the modified New York criteria, which require bilateral grade ≥2 or unilateral grade ≥3 sacroiliitis for a diagnosis of AS. The modified Stoke Ankylosing Spondylitis Spinal Score (mSASSS) provides a quantitative measure of spinal structural damage (63). The critical and well-documented limitation of conventional radiography is that it detects structural damage—erosions, sclerosis, and ankylosis—rather than active inflammation, and radiographic changes typically require 5–10 years of disease activity to become reliably detectable. Inter-reader reliability is moderate at best, with kappa values for sacroiliitis grading ranging from 0.4 to 0.7 (10, 64). In nr-axSpA, radiography is, by definition, negative, and its role is limited to excluding other pathologies rather than confirming the diagnosis.

### 4.2 MRI: sensitive but not specific

MRI has transformed early axSpA diagnosis by enabling visualization of active inflammation, specifically, bone marrow edema (BME) on STIR or T2-weighted fat-suppressed sequences, years before structural damage became radiographically apparent. The ASAS definition of a positive MRI (≥2 BME lesions on the same slice or ≥1 lesion on ≥2 consecutive slices, in a pattern highly suggestive of SpA) provides a standardized framework for interpretation (65). The SPARCC (Spondyloarthritis Research Consortium of Canada) scoring system enables reliable quantification of SIJ inflammation, with intraclass correlation coefficients consistently exceeding 0.80, even after minimal reader calibration (66). The STIR technique and the SPAIR T2w sequence showed high agreement in evaluating sacroiliac joint subchondral bone marrow edema in patients with SpA (67).

The diagnostic Achilles’ heel of sacroiliac MRI is specificity. BME in the sacroiliac joints is not pathognomonic for axSpA; it occurs with substantial frequency in healthy populations—approximately 17–25% of healthy individuals over age 30 meet ASAS criteria for a positive MRI, rising to approximately 41% in elite athletes and approximately 60% in postpartum women (68). Structural lesions, including erosions and fat metaplasia, also increase with age, with erosions detected in approximately 20% of healthy subjects overall and 39% of those aged 40 or older (68). To address this limitation, the updated ASAS definitions now formally encompass structural MRI lesions—including erosions, fat metaplasia, backfill, and ankylosis—which offer complementary diagnostic value beyond BME, particularly in nr-axSpA (69). These findings have prompted a growing emphasis on interpreting MRI results within the appropriate pre-test probability context, with the understanding that a positive MRI in a low-risk patient (e.g., a young athlete with mechanical back pain) carries far less diagnostic weight than the same finding in a patient with typical IBP, HLA-B27 positivity, and elevated CRP.

### 4.3 CT: high specificity for structural disease

Computed tomography (CT) provides superior visualization of structural changes in the sacroiliac joints compared with both radiography and MRI. A landmark 2022 head-to-head comparison of 163 patients demonstrated CT specificity of 97.3% and inter-rater reliability (κ = 0.875), both exceeding the corresponding values for MRI (specificity = 86.5%, κ = 0.665) and radiography (specificity = 67.6%, κ = 0.517) (10). Low-dose CT protocols now deliver effective doses as low as 0.42mSv (70) while achieving 76% sensitivity for sacroiliitis detection, outperforming the X-ray sensitivity of 66% (10). CT cannot assess bone marrow edema and therefore cannot detect purely inflammatory sacroiliitis without structural change, which remains MRI’s domain. The combined CT-plus-MRI approach, achieving diagnostic accuracy of 93% in a 2020 study, leverages the complementary strengths of both modalities (71).

### 4.4 Ultrasound: an adjunctive role

Musculoskeletal ultrasound enables radiation-free, real-time detection of peripheral enthesitis, synovitis, and tenosynovitis; Power Doppler Ultrasound (PDUS) further quantifies entheseal microvascular hyperemia as a marker of active inflammation, in accordance with OMERACT consensus criteria (72, 73). DEUS cohort research confirmed PDUS signals as the most specific feature distinguishing axSpA inflammatory enthesitis from degenerative lesions (OR = 8.77) (74). Regrettably, ultrasound cannot visualize sacroiliac joint pathology or MRI-characteristic bone marrow edema, so meta-analyses only support its auxiliary value for sacroiliitis screening (75). Serial PDUS objectively measures reduced entheseal hyperemia after TNF/IL-17 inhibitor therapy (76). Still, severe operator variability and poor reproducibility in mild inflammatory lesions preclude PDUS from serving as a standalone diagnostic for axSpA.

### 4.5 The human-reader ceiling

A cross-cutting limitation of all conventional imaging modalities is their dependence on subjective human interpretation. Inter-reader variability, even among experienced musculoskeletal radiologists, introduces noise into the diagnostic signal that no improvement in imaging hardware can eliminate. This human-reader ceiling is the bottleneck that radiomics and machine learning methods are explicitly designed to address—not by replacing radiologists, but by providing objective, quantitative imaging biomarkers that augment and standardize human interpretation.

## 5 Imaging biomarkers in the AI era: machine learning

### 5.1 The radiomics pipeline

Radiomics refers to the high-throughput extraction of quantitative imaging features from medical images—features that may capture tissue properties invisible to the human eye. The standard radiomics pipeline involves region-of-interest (ROI) segmentation of the sacroiliac joints, followed by extraction of shape descriptors, first-order histogram statistics, and textural features derived from gray-level co-occurrence (GLCM), run-length (GLRLM), and size-zone (GLSZM) matrices, often augmented by wavelet-transformed images. Feature selection—via LASSO regression, minimum redundancy maximum relevance (mRMR), or Boruta algorithms—reduces dimensionality before model building (77). The Image Biomarker Standardization Initiative (IBSI) provides reference standards for radiomic feature computation, though IBSI compliance remains poorly reported in axSpA studies to date (78).

### 5.2 Traditional machine learning for axSpA diagnosis

The integration of radiomic features with classical machine learning classifiers represents the most established paradigm for quantitative imaging biomarker development in axSpA. Across multiple imaging modalities, radiomics has demonstrated robust diagnostic performance. On CT, Castro-Zunti et al. achieved an AUC of 0.97 for erosion detection using GLCM/LBP features with random forest and k-NN classifiers (79). In an MRI study, Tenório et al. first showed that textural features could discriminate SpA-related sacroiliitis from controls (AUC ≥ 0.80) and distinguish axial from peripheral subtypes (80, 81). Faleiros et al. achieved 100% sensitivity and 95.6% specificity using an MLP classifier with only six features to classify active sacroiliitis (82). Kepp et al. demonstrated that texture analysis outperformed qualitative assessment in differentiating inflammatory from degenerative sacroiliac changes (83). Venerito et al. applied XGBoost to BME radiomic features, achieving an AUC of 0.77 for discriminating axSpA from axial psoriatic arthritis (84). A 2025 study further reported that texture analysis surpassed radiologists in classifying healthy controls, nr-axSpA, and r-axSpA, with T1WI- and T2WI-based models achieving AUCs of 0.934/0.917 and 0.976/0.848, respectively (85). Zheng et al. demonstrated that radiomics-based BME assessment (AUC = 0.90) correlates highly with SPARCC scores, and that T1WI-based radiomics detects structural lesions with expert-level performance (AUC = 0.82) (77, 86).

Overall, TML algorithms for sacroiliitis feature discrimination on MRI reported AUC values ranging from 0.75 to 0.98 across studies, with performance dependent on feature selection methodology, sample size, and the specific classification task (87).

### 5.3 Deep learning: end-to-end imaging biomarker discovery

Deep learning bypasses the explicit feature-engineering step in classical radiomics by learning hierarchical feature representations directly from image data. In the axSpA imaging literature, convolutional neural networks (CNNs) have been the dominant architecture, applied across MRI, CT, and X-ray, covering three major task types: classification/detection, lesion segmentation, and grading/scoring.

Classification and detection identify sacroiliitis and distinguish axSpA from controls or degenerative changes. On X-ray, Bressem et al. achieved validation/test AUCs of 0.97/0.94 with ResNet-50 (κ = 0.79/0.72) (88); Üreten et al. reported 89.9% accuracy and 90.9% sensitivity with VGG-16 (89). On CT, Van Den Berghe et al. developed a U-Net + dual-CNN pipeline for erosion (AUC = 0.92) and ankylosis (AUC = 0.91) detection (90); Xu et al. reported external accuracies of 87.1–89.6% and AUCs of 0.88–0.93 in 1,590 multi-center patients (91). On MRI, Bressem et al. achieved AUCs of 0.94 for active inflammation (sensitivity 88%, specificity 71%) and 0.89 for structural changes (85%, 78%) (92); Bordner et al. achieved an AUC up to 0.98 and a MCC of 0.90 (93, 94); Lee et al. reported a patient-level sensitivity of 94.7%, a specificity of 69.1%, and an AUC of 0.816 (95). Lesion segmentation enables pixel- or voxel-level identification of pathological changes, serving as the foundation for automated quantitative assessment. On CT, Zhang et al. achieved a Dice coefficient of 0.889 for SIJ segmentation using nnU-Net (96). On MRI, Rzecki et al. and Ózga et al. developed fully automatic BME segmentation algorithms, with the latter achieving a remarkable Dice coefficient of 0.98 (97, 98). Grading and scoring quantify disease severity or specific imaging changes to support progression assessment and treatment decisions. On X-ray, Koo et al. automated vertebral body corner grading (0–3 scale) using HRNet + ResNet-152, achieving 93.7% sensitivity and 95.7% accuracy for grade 3 lesions—providing proof-of-concept for mSASSS automation (99); Dorfner et al. showed that anatomy-centered preprocessing (cropping to SIJ bounding box) improved generalizability, with external AUCs of 0.899, 0.846, and 0.957 (100).

However, deep learning suffers from several limitations, including substantial data requirements, insufficient interpretability, and poor model stability in small sample axSpA cohorts. In contrast, conventional radiomics can output quantitative features with clear anatomical interpretability, demonstrate robust performance under limited sample conditions, and operate solely on standard CPUs. The emerging hybrid deep learning radiomics (DLR) framework, which integrates auto-learned deep features from CNNs with handcrafted radiomics and clinical features into a traditional machine learning classifier, has gained considerable attention in oncological imaging (101, 102). By combining deep learning’s representational power with radiomics’ robustness in small samples and interpretability, DLR offers a balanced approach. Two published studies on axSpA have validated the superiority of hybrid models: Zhang et al. reported an AUC of 0.91 for their DLR ensemble model, significantly outperforming the pure radiomics model (AUC 0.868) (103); Xie et al. developed a multi-center KNN-based hybrid model that maintained a stable AUC range of 0.85–0.912 (104).

### 5.4 The data chasm

**Table 2** compares radiomics biomarkers with Deep learning-derived biomarkers from different dimensions. The most critical limitation in axSpA AI research is methodological rather than technical, affecting both radiomic and deep learning biomarkers. Most published studies have median sample sizes below 200 patients, are single-center and retrospective, and include external validation in fewer than 20% (87). Data leakage—where slices from the same patient appear in both training and test sets—has been documented in sacroiliitis studies. Compliance with reporting and evaluation frameworks—including CLAIM, TRIPOD+AI, STARD/STARD-AI, QUADAS-AI, PROBAST+AI, and IBSI —remains inconsistent and lags behind technical progress. Specific methodological risks that require explicit reporting include inadequate segmentation reproducibility, failure to harmonize scanner effects and multi-center protocol variations, and unclear reference standards (78, 105, 106). Additionally, most structural lesion detection studies lack histopathological validation, relying on expert consensus as the reference standard despite only moderate inter-reader agreement—thereby introducing reference-standard bias. While these challenges are not unique to axSpA, they carry weight here because the cost of missed diagnosis is high: years of untreated inflammation and irreversible structural damage. The gap between reported AUCs and the reliability required for clinical decision-making remains the central tension of the field.

**Table 2:** Comparison of different machine learning-derived biomarkers.

Evaluation dimension	Radiomics biomarker	Deep learning-derived biomarker
Feature Source & Definition​	IBSI-standardized mathematical features (histogram, GLCM/GLRLM/GLSZM texture, morphology.	Auto-learned high-level latent representations from raw pixels (CNN/ViT), requiring no explicit mathematical formulas.
Interpretability & Regulatory Compliance​	Features carry clear mathematical, anatomical, and pathological semantics; clinicians place great trust in them.	Deep latent features lack a fixed biological interpretation; they rely solely on *post hoc* visualization (e.g., Grad-CAM), which is susceptible to noise.
Data & Computational Requirements​	Stable modeling with hundreds of samples; runs on standard CPUs without high-end hardware—suited for size-limited diseases such as axSpA.	Data-hungry, small-sample overfitting; needs large, labeled datasets, high-end GPUs, and standardized multi-center data.
Dependence on ROI Segmentation​	Requires precise ROI segmentation, with inter-observer variability introducing variance—but this forces model focus on biologically relevant regions; partially mitigated by nnU-Net-based automatic segmentation.	End-to-end weakly supervised learning reduces the manual segmentation burden but risks learning irrelevant shortcuts (e.g., bowel gas, positioning artifacts) in the absence of anatomical constraints.
Detection Sensitivity for Early Subtle Lesions​	Only captures predefined imaging information; limited sensitivity for micro-erosion in nr-axSpA.	Captures subtle inflammation beyond human/radiomics detection; enhances early BME detection in nr-axSpA.
Standardization & Multi-Center Reproducibility​	IBSI-standardized; fixed PyRadiomics/LIFEx pipeline ensures high comparability across hospitals and scanners; compatible with multi-center registries (e.g., ACR RISE); stable modeling with moderate samples (hundreds).	No unified standard; features across different networks (VGG, ResNet, 3D U-Net) are incompatible; AUC degrades in multi-center validation; results cannot be directly cross-compared.
Clinical Deployment Cost & Implementation Barriers​	Standard-CPU batch extraction, small model footprint, fast inference; directly embeddable into PACS; rapid grassroots deployment.	Training demands GPU clusters; deployment needs dedicated workstations and complex maintenance. Currently limited to large medical centers.
Privacy Compliance & FAIR Data Alignment​	Low-dimensional, small data footprint; ideal for federated learning (feature weights only, no raw images); privacy-compliant and FAIR-aligned.	Large model parameters; federated learning requires heavy gradient transmission, raising privacy risks; lacks unified metadata—FAIR-incompatible for reusability/interoperability.
Overall Clinical Translation Positioning​	Optimal short-term clinical option for axSpA diagnosis/stratification, and multi-center validation; easier regulatory pathway.	Mid-to-long-term precision medicine core; suitable for early screening, prognosis, and full automation.

### 5.5 Explainability: making imaging biomarkers clinically transparent

Explainability methods, including Grad-CAM, SHAP, and LIME, have been applied to SIJ AI models to visualize the regions that drive decisions (104). However, *post-hoc* explanations can be unstable and lack standardized evaluation (77, 107). In axSpA, explainability serves a dual purpose. First, it provides radiologists with visual confirmation that the model focuses on clinically relevant anatomy (subchondral bone, bone marrow, joint space) rather than artifacts or extraneous structures. Second, quantitative feature attribution maps (e.g., SHAP values) can themselves serve as novel imaging biomarkers—providing spatially localized diagnostic information beyond qualitative reading. In the deep radiomics paradigm, Grad-CAM heatmaps from the CNN’s final convolutional layer visualize where the disease signal is strongest. However, current explainability methods in axSpA have not undergone rigorous prospective evaluation with clinician end-users, and their regulatory role remains undefined. The transition from black-box prediction to explainable diagnosis is essential for clinical trust, but reliable, scalable methods for achieving this transition are still under development.

## 6 Toward multi-modal integration

### 6.1 The case for fusion

**Table 3** summarizes the different modalities of axSpA biomarkers reviewed in the preceding sections, and each modality confronts an inherent ceiling. Clinical markers lack sensitivity, particularly in HLA-B27-negative and nr-axSpA patients. Molecular markers, while mechanistically informative, have not yet achieved standalone diagnostic accuracy sufficient for clinical use. Conventional imaging is limited by human subjectivity, and AI-enhanced imaging, while addressing that subjectivity, analyzes pixels in isolation from the biological context that gives those pixels their clinical meaning. The logical response to these individual ceilings is integration. A patient whose SIJ MRI is equivocal may have a metabolite profile that tips the diagnostic balance; a patient who is HLA-B27-negative may have imaging features that, when quantified by radiomics, exceed the diagnostic threshold. Multi-modal fusion—the integration of clinical, molecular, and imaging biomarkers into a unified diagnostic model—exploits the orthogonality of information across modalities and, in principle, represents the most powerful diagnostic strategy.

**Table 3:** Summary of axSpA biomarkers.

Biomarker modalities		Key markers/Tools	Diagnostic strength	Key limitation	Clinical readiness
Clinical		HLA-B27, IBP criteria, CRP	Established; high sensitivity (HLA-B27)	Low specificity in low-prevalence settings; ethnic variation	Routine clinical use
Molecular	Genetic	ERAP1, IL23R, PRS panels	Mechanistic insight; PRS > HLA-B27 alone	Cross-ethnic portability limited	Research only
	Proteomic	Multi-analyte panels (Olink, SomaScan)	Combinatorial signatures outperform single markers	No validated diagnostic panels; replication lacking	Research only
	Metabolomic	Tryptophan metabolites, SCFAs	Biologically plausible; gut-joint axis link	Cost, accessibility; no clinical recommendations	Discovery phase
	Liquid Biopsy	miR-146a/155/29a panels, exosomes	Non-invasive; multi-marker AUC ~0.91 (small cohorts)	Small single-cohort studies; no independent validation	Discovery phase
Imaging	Conventional	X-ray, MRI (STIR), CT (low dose)	MRI sensitivity ~82%; CT specificity ~97%	MRI: false positives; X-ray: low sensitivity	Routine clinical use
	AI	Radiomics, TML, DL	AUC 0.75–0.97; matches general radiologists	No external validation in most studies; no deployment pathway	Research (approaching clinical readiness)
Multi-modal Fusion		Clinical + imaging + molecular integration	Conceptually strongest; AUC gain ~0.04 (early evidence)	No benchmark datasets exist; empirical evidence is thin	Conceptual/early research

### 6.2 axSpA-specific fusion studies

The literature on multi-modal fusion in axSpA diagnosis remains sparse. Constrained by the limited availability of validated molecular biomarkers in the field—beyond HLA-B27, no other routine diagnostic molecular markers have been established—existing fusion models have only incorporated radiomic features, deep learning radiomics (DLR) features, and basic clinical parameters. Several studies have demonstrated that combining quantitative imaging features with clinical variables such as HLA-B27 and CRP significantly improves diagnostic AUC, confirming that even basic data fusion can yield substantial clinical gains (92, 104, 108). Imaging-genomics association studies have linked SIJ bone marrow edema severity on MRI to IL23R genotype, whereas associations with ERAP1 were not significant after Bonferroni correction (109). However, existing studies have not yet explored the correlation between pathogenic genes and radiomic biomarkers, nor have they developed an integrated multi-modal diagnostic model that combines genomic, radiomic, and clinical information. Significant gaps, therefore, remain in fusion research in this area (110).

### 6.3 The infrastructure gap

The rate-limiting step for multi-modal axSpA diagnostics is not algorithmic. Adequate fusion methods exist and have been validated in oncology (TCGA) and neurology (ADNI), where large public multi-modal datasets have been available for over a decade (111). Rheumatology—and axSpA specifically—lacks comparable infrastructure. Building such a resource, a prospective multicenter cohort with matched clinical, genomic, proteomic, and metabolomic data, plus standardized imaging data and longitudinal follow-up, would require substantial investment and multi-institutional coordination. Lessons from oncology and neurology are instructive: TCGA and ADNI have demonstrated that shared, standardized, multimodal cohorts can accelerate biomarker translation by an order of magnitude compared with fragmented, single-institution efforts. These precedents suggest that the infrastructure investment—while substantial—is a proven model with considerable returns in other disease domains. However, the fundamental constraint for axSpA remains: the algorithms exist; the data does not.

## 7 Challenges, future directions, and conclusions

### 7.1 A clinical-utility framework for emerging biomarkers

No individual genetic, laboratory, molecular, or imaging biomarker can currently independently diagnose or exclude axSpA. Therefore, emerging biomarkers should be assessed not only by statistical performance, such as AUC, but also by their ability to improve clinical decision-making. A clinically useful biomarker should be reproducible across platforms and settings, distinguish axSpA from relevant mimics, and provide incremental value beyond clinical assessment, HLA-B27, inflammatory markers, and imaging. Importantly, it should reduce diagnostic delay, improve confidence in equivocal cases, and limit unnecessary testing or misdiagnosis. AI-based and multimodal approaches should be developed as decision-support tools rather than replacements for clinical judgment. Their value should be demonstrated through external validation, prospective studies, and assessment of feasibility, cost-effectiveness, and applicability across diverse populations. Diagnostic utility should also be distinguished from treatment-response utility, as biomarkers useful for diagnosis may not necessarily predict treatment outcomes.

### 7.2 Validation: the rate-limiting step

Across all three biomarker modalities, the transition from discovery to clinical validation represents the most significant bottleneck. For AI-based imaging biomarkers, fewer than 5% of FDA-cleared radiology AI devices have undergone prospective testing (112). For molecular biomarkers, multi-center external validation cohorts are the exception rather than the rule. The 2025 Chinese guideline on axSpA biomarkers concluded that no metabolomic or genetic markers (other than HLA-B27) currently meet the evidence threshold for routine use, underscoring the critical gap between discovery and validation (55). For multimodal fusion models, prospective clinical utility trials that demonstrate not just that a biomarker predicts diagnosis but that its use changes clinical decisions and improves patient outcomes do not yet exist in axSpA. The SPARCC, SPACE, and DESIR cohorts provide established platforms for biomarker validation. Still, they were not designed as multi-modal diagnostic cohorts and lack the integrated molecular phenotyping that the fusion paradigm requires (87).

### 7.3 Standardization

Standardization gaps affect every layer of the diagnostic biomarker pipeline. Radiomic feature computation remains inconsistently aligned with IBSI reference values (78). Biomarker cutoff values—whether for anti-CD74 antibody titers or radiomic signature scores—vary across studies, lacking a consensus framework for harmonization. AI model evaluation protocols are heterogeneous, making cross-study comparison unreliable. Reporting guideline adherence—TRIPOD-AI for prediction models, CLAIM for radiomics, STARD for diagnostic accuracy studies—is inconsistent (78). These are addressable problems, but their adoption in axSpA research lags the technical pace of discovery.

### 7.4 Clinical translation and deployment

The gap between a published model and a deployed clinical tool is wide and often underappreciated. Regulatory clearance pathways differ between the United States (FDA 510(k) pathway for SaMD, now augmented by Predetermined Change Control Plans enabling adaptive learning) and the European Union (EU AI Act imposing risk-stratified conformity assessment and post-market surveillance) (107, 112). Beyond regulatory frameworks, clinical practice guidelines—including the EULAR recommendations for axSpA management and the ASAS consensus definitions for MRI—provide complementary guidance for the clinical application of biomarkers and imaging in routine practice.

A further barrier, rarely discussed in the axSpA biomarker literature, is cost-effectiveness. A multimodal diagnostic pathway incorporating HLA-B27 genotyping, a proteomic panel, STIR MRI of the sacroiliac joints, and AI-assisted image interpretation would incur substantially higher per-patient costs than current diagnostic workflows based on clinical assessment, CRP, and selective imaging. In publicly funded healthcare systems, adoption of such pathways requires not only demonstration of clinical validity but also a formal health-economic evaluation, typically a cost-per-quality-adjusted-life-year (QALY) analysis, and no such evaluation has been conducted for any multimodal axSpA diagnostic strategy to date. The net result is that models that perform well in the literature have no established path to the clinic, and the economic case for deploying them has not been made.

### 7.5 Fairness, bias, and generalizability

HLA-B27 prevalence varies more than tenfold across ethnic groups. AI models trained predominantly on European-ancestry populations—as the majority of published axSpA imaging AI studies are—may fail when applied to Asian, African, or Hispanic patients, in whom the relationships among genetic background, imaging phenotype, and disease risk differ (9, 113). A 2024 analysis of FDA-approved AI/ML medical devices found that race and ethnicity data were reported in only 3.6% of submissions, and socioeconomic data in 0.9% (112). A separate study demonstrated that an FDA-cleared, CE-marked commercial AI tool for knee osteoarthritis grading exhibited 13.6% non-uniformity between left and right knee images of the same patient when images were horizontally flipped—evidence that bias can be “built into regulatory-approved commercial AI tools” (114). These findings carry a specific warning for axSpA: a diagnostic AI that performs well in a Northern European cohort (HLA-B27 prevalence ~15%, B*27:05 dominant) may not perform similarly in a Japanese cohort (HLA-B27 prevalence ~1%, different subtype distribution) or an African American cohort (HLA-B27 prevalence ~2%, AS patients only ~48% HLA-B27). Fairness-aware machine learning methods—ensemble debiasing, adversarial debiasing, resampling, and reweighting—offer technical mitigation strategies, but their application in axSpA AI research remains rare (113).

### 7.6 Conclusions

The axSpA diagnostic biomarker landscape has transformed over the past decade. Clinical biomarkers (HLA-B27, IBP criteria) provide a well-validated but insufficient foundation. Molecular biomarkers—genomics, proteomics, metabolomics, liquid biopsy—are expanding the toolkit, with the key trend shifting from single molecules to multi-analyte panels. However, independent validation remains the critical unmet need. Imaging biomarkers have evolved most dramatically: conventional modalities remain the clinical workhorse, while AI-driven radiomics and deep learning now match or approach expert performance for sacroiliitis detection, grading, and quantification.

Three conclusions emerge. First, no single modality is sufficient - each provides orthogonal diagnostic information, and the ceiling of each approach is now well characterized. Second, AI-driven imaging is the fastest-moving frontier, but translation is constrained not by model performance but by the lack of prospective validation, standardization, and deployment infrastructure. Third, biomarker discovery and infrastructure construction are complementary priorities: novel molecular candidates require further validation, while the datasets and frameworks for integrating validated biomarkers must be built simultaneously. The era of single-modality research has produced a rich toolkit; the task ahead is to refine it through rigorous validation and build the integrative infrastructure—multi-modal datasets, standardized reporting, and prospective trials—needed for clinical deployment.
